# Tomorrow’s World: Is Digital Health the Disruptive Innovation that will Drive the Adoption of Integrated Care Systems?

**DOI:** 10.5334/ijic.4638

**Published:** 2018-12-27

**Authors:** Nick Goodwin

**Affiliations:** 1 IFIC, Wolfson College, Linton Road, Oxford, GB

**Keywords:** Digital health, implementation science, innovation, integrated care, technology

As a teenager I was drawn to programmes like BBC’s *Tomorrow’s World* that looked at the changing face of technology and sought to predict the future of science [1]. Many of the pioneering technologies featured in the 1960s and 1970s are now common practice, for example: kidney dialysis, heart transplant surgery, laser eye surgery, personal/tablet computers, and the Internet of Things. However, most featured innovations simply failed to catch on or were superseded by other disruptive innovations – for example, in the way digitised information is now stored and used for multiple purposes and the methods through which such information can be communicated.

The reason for highlighting this childhood television programme is to recognise the sheer pace at which innovations in digital health and care have progressed in such a short space of time. My own connection to recent research looking at the use of telehealth and telecare technologies within large field trials, and in EU-funded studies, demonstrates that technologies become outdated even during the lifetime of a 3 to 4-year evaluation programme [2,3,4,5]. Most of the approaches to home-based telehealth that sought to monitor and manage people’s chronic illnesses in these studies are already looking like first-generation mobile phone technology – clunky, impersonal, immobile. They were simply limited in their capabilities compared to the simplicity of today’s tablet-based solutions, mobile devices, wearables and even ingestible devices that can continuously collect data to monitor symptoms, activity and outcomes and provide personalised feedback on a person’s wellbeing. Cloud storage, blockchain technology, cognitive computing and artificial intelligence are enabling new data analytics in ways previously unimagined.

The role of digital health solutions, and the promotion of an industry that can support it, has become a key policy goal across Europe and other countries – for example in the push to achieve a digital single market [6]. The idea is that disruptive digital innovations can play a significant role in curbing the long-term rise in the costs of health and care, empower and engage service users, and enable better care outcomes and experiences. Key to this is the more accurate and timely identification of diseases and risks, and the potential to use technology to shift care towards home-based solutions that enable self-management and preventative action, thus promoting both efficiency and effectiveness in care provision.

In many ways, the strategy has similar objectives to the integrated care movement in the need to design and implement new ways of care delivery [7]. Indeed, the role of information, communication and technology is commonly regarded as one of the essential ingredients in enabling the success of integrated care [8]. It has the most uncommon dual property in this regard. It is simultaneously the grease that allows integrated care systems to operate as smoothly as they can through good communication of information between care professionals and services users, but it is also the glue that binds care systems together. For example, it potentially enables a golden thread to exist that links the performance and funding systems devised at a macro-level to help influence the behaviours and motivations that drive high quality person-centred and coordinated care at the micro-level.

Adoption of these emerging technologies, while undoubtedly favourable, will require a high level of coordination and interoperability across the various sectors of care to ensure that the potential benefits can be realised. However, it is at this point that the integrated care movement is faced with its most pressing problem. Whilst digital innovations emerge at pace, systemic reform in the way health and care is designed and delivered is glacial by comparison (if at least not geological!). Dual innovation is needed since if care providers and organisations remain unwilling to work in networks and teams that deliver more integrated and preventative care in community and home-based settings then the overlay of technology is likely not to have the desired effect.

As Lewis and Goodwin (2017) concluded when developing implementation guidance as part of the recently concluded CareWell project, technology-enabled integrated care must be recognised as a complex service innovation that requires strong stakeholder relationships at all levels to facilitate the change in co-producing the design, implementation and mainstreaming of new service models and delivery systems [9]. Rather than a blinkered focus on digital health innovation for its own sake, this suggests that real disruptive innovation will only come through effective change strategies that influence, educate, train and enable transformation in the way care professionals work and how they engage effectively with patients and carers. Only this will ensure that implementation of integrated care at the clinical and service level (where it matters most) can be fully effective.

For this to happen, some simple rules may need to be observed in the use of technology [10]:

- keep it simple for patients and carers to use, and for professionals to adopt;
- tailor the service to the specific needs of the user with digital solutions which can flex in line with their health and wellbeing; consider how they might best use and accept new technology;
- enhance human contact by better connecting patients to family, friends and care professionals; users must feel safe, secure and empowered;
- embed an IT infrastructure to act as the bedrock of better care through integrated information systems; and
- build relationships and networks to influence behaviours, build alliances, and overcome the significant mismatch of motives that exist between patients, carers, professionals, commissioners and industry.

In conclusion, integrated care will not evolve efficiently without the technological tools to support it. However, dual innovation is required that enables transformational change to happen in the way care providers work together to achieve the best outcomes for people and populations. For me, the latter is the disruptive innovation that will be required if integrated care is to prove successful with technology providing the tools through which to facilitate it.
